# Degradation of Exogenous Fatty Acids in *Escherichia coli*

**DOI:** 10.3390/biom12081019

**Published:** 2022-07-22

**Authors:** Viola Pavoncello, Frédéric Barras, Emmanuelle Bouveret

**Affiliations:** Stress Adaptation and Metabolism Unit, Department of Microbiology, Université Paris-Cité, CNRS UMR6047, Institut Pasteur, 75015 Paris, France; vio.pavoncello@pasteur.fr (V.P.); frederic.barras@pasteur.fr (F.B.)

**Keywords:** fatty acids, beta-oxidation, *fad* genes, *Escherichia coli*, FadR

## Abstract

Many bacteria possess all the machineries required to grow on fatty acids (FA) as a unique source of carbon and energy. FA degradation proceeds through the β-oxidation cycle that produces acetyl-CoA and reduced NADH and FADH cofactors. In addition to all the enzymes required for β-oxidation, FA degradation also depends on sophisticated systems for its genetic regulation and for FA transport. The fact that these machineries are conserved in bacteria suggests a crucial role in environmental conditions, especially for enterobacteria. Bacteria also possess specific enzymes required for the degradation of FAs from their environment, again showing the importance of this metabolism for bacterial adaptation. In this review, we mainly describe FA degradation in the *Escherichia coli* model, and along the way, we highlight and discuss important aspects of this metabolism that are still unclear. We do not detail exhaustively the diversity of the machineries found in other bacteria, but we mention them if they bring additional information or enlightenment on specific aspects.

## 1. Introduction

Fatty acids (FA) are the principal components of the membrane structures of all living organisms and play essential roles in membrane architecture, homeostasis, and transport. Therefore, FA metabolism occupies a central position in the microbial metabolome, and the pathways involved are conserved between prokaryotes and eukaryotes.

FAs are carboxylic acids (R-COOH) with a long aliphatic chain that can be either saturated or unsaturated. FAs are classified based on the length of the aliphatic chain: short-chain fatty acids (SCFAs) are made of less than 6 carbons, medium-chain fatty acids (MCFAs) are made of 6 up to 10 carbons, and long-chain fatty acids (LCFAs) are made of 12 carbons or more. The aliphatic chain length determines the mode of entry of FA within the cell and the machinery of degradation involved. To be oxidized, the M/LCFAs require the activation of the fatty acid degradation (*fad*) regulon, the main object of this review. Full degradation of SCFAs requires the Ato machinery.

*Escherichia coli* has been used as the prototypical prokaryote for studying FA degradation for over 50 years. Thanks to the ease of genetic manipulation, many features of FA catabolism have been disclosed. However, the key role of FA degradation factors in adaptation to stress and in virulence has been evidenced more recently in many pathogenic bacteria [1,2,3,4].

In addition, the explosion of genomic sequence information provided complete genomic data sets for many organisms, highlighting parallels and gaps among bacterial species, and the more complex eukaryotic FA degradation. Similarities between prokaryotes and eukaryotes include (i) the conservation of enzyme structures and functions (i.e., most metabolic enzymes share 30–40% identical amino acids), (ii) the conserved reaction mechanisms as well as the common enzymatic pathways, which reflect the sequence conservation; (iii) high-affinity transport systems for the uptake of exogenous FAs. In addition, the degradation of FAs in eukaryotes is controlled by transcriptional regulators sensing FAs in a manner very similar to the transcriptional regulation of FA degradation (*fad*) genes in response to LCFAs in prokaryotes [5].

In this review, we present an up-to-date description of FA degradation in *E. coli*, from the description of the paradigmatic FA β-oxidation cycle to the complexity of the transcriptional regulation orchestrating expression of the *fad* genes in response to different environmental and nutrimental conditions. We mention features observed in other bacteria if they are important to highlight or discuss specific aspects. In addition, we discuss the importance of redundancy of the Fad enzymes in bacteria, and specifically the role of homologs to the canonical fatty acid degradative (Fad) enzymes in *E. coli*. We conclude the review with open questions that we think are important to study in order to better understand this central metabolism of the bacterial cell.

## 2. Pathways of Fatty Acid Degradation

Fatty acids (FAs) are important sources of both metabolic energy and essential components of membranes in all organisms. In bacteria, FAs are mainly found as components of the phospholipids (PLs), in which two FAs are attached to a glycerol backbone. In Gram-negative bacteria, PLs make up the two leaflets of the bacterial inner membrane (IM) and the internal leaflet of the outer membrane (OM). In the external monolayer of the OM, FAs also constitute the hydrophobic part of the lipopolysaccharide (LPS). PLs and LPS are constantly synthesized, modified, and destroyed to maintain membrane homeostasis and to respond to environmental stressors. Free FAs can be released during these processes, serving as signaling molecules for the regulation of the LPS levels or providing an extra carbon source when other sources are depleted [2,6,7]. Nonetheless, the main source of free FAs remains the surrounding environment. Especially in the gut environment, the dietary intake of the host (i.e., LCFA from dietary triglycerides) as well as the S/MCFA byproduct from the microbiota metabolism provide an important carbon source for the bacterial gut inhabitants. This ability to use FAs as sole carbon and energy sources in aerobic and anaerobic environments allows bacteria to adapt to specific niches in mammalian hosts and to escape the competition by occupying alternative niches.

### 2.1. Translocation of Fatty Acid in the Cell

**Figure 1:**
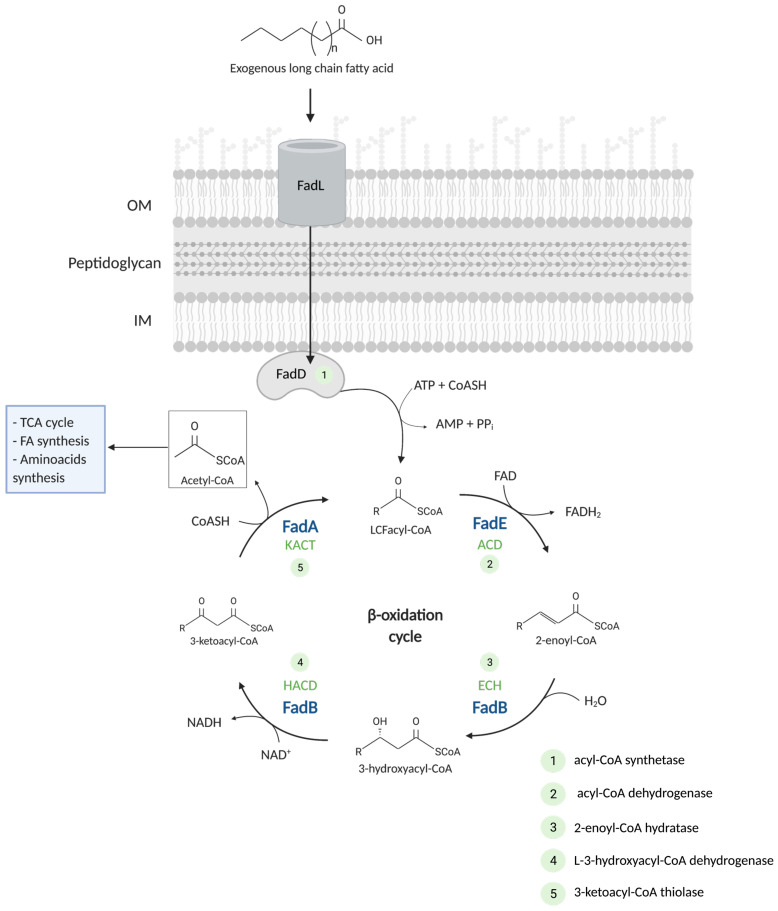
The β-oxidation pathway of fatty acid degradation. Exogenous long-chain fatty acids are imported across the cell membranes via a transport/acyl-activation mechanism involving the outer membrane protein FadL and the inner membrane-bound acyl-CoA synthetase FadD (1). Upon activation, the fatty acyl-CoA enters the β-oxidation cycle. Four enzymatic reactions follow: a first oxidation by FadE, generating one FADH_2_ molecule (2, ACD), and three reactions carried out by the tri-functional complex FadBA: a hydration (3, ECH), a second oxidation generating a NADH molecule (4, HACD), and a final thiolytic cleavage (5, KACT), producing a molecule of acetyl-CoA. The acetyl-CoA is the substrate for key metabolic processes of the central metabolism. The enzymes of the β-oxidation cycle are shown in blue. Numbers correspond to the five reactions required for the activation and catabolism of FA. ACD = acyl-CoA dehydrogenase; ECH = enoyl-CoA hydratase; HACD = hydroxy-acyl-CoA dehydrogenase; KACT = ketoacyl-CoA thiolase.

Whereas medium- (C_7_–C_11_, MCFA) and short-chain fatty acids (C_4_–C_6_, SCFA) may enter the cell by free diffusion or via porins, specialized importers are required to uptake exogenous LCFAs (>C_12_) [8]. In *E. coli*, the entrance of LCFAs into the cell consists of their import through the outer membrane by the specialized OM transporter FadL, followed by crossing of the inner membrane coupled to FA activation by the cytosolic acyl-CoA synthetase FadD (Figure 1).

**Figure 2:**
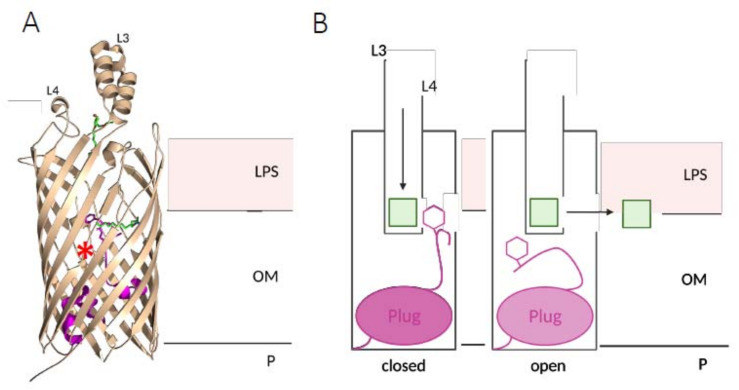
(**A**) FadL structure. The extracellular loops L3 and L4 are indicated. The N-terminal hatch domain (the plug, in panel **B**) and the phenylalanine 3 (the hexagon) are in pink. Two fatty acid molecules are shown in green, in the different binding sites: the low-affinity binding site at the entrance of the channel (on the top) and the high-affinity binding site in the channel (on the bottom). The red star shows the lateral opening in the β-barrel. (**B**) Model for ligand-gated transport in FadL. This model has been proposed in [9]. FadL is depicted as the black rectangle. In the closed state (**left**), the LCFA substrate (green square) binding to the high-affinity site in the channel causes a conformational change in the N-terminal hatch domain. The resulting displacement of the phenylalanine 3 (pink hexagon) leads to the open state of FadL (**right**) and to substrate release. The LCFAs are released in the OM through the lateral opening in the barrel wall. The putative positions of the outer membrane (OM) boundaries are indicated with horizontal lines, with the lipopolysaccharide (LPS) extracellular side at the top and the periplasm (P) at the bottom. This and the following figures were made with PyMOL.

FadL is a 48 kDa β-barrel protein composed of 14 β-strands, with two extracellular loops, L3 and L4 [9] (Figure 2). An exposed hydrophobic groove extends from the extracellular surface of the protein to an opening in the barrel wall between two β-strands, without connecting the extracellular milieu with the periplasm. Finally, the N-terminal hatch domain of FadL plugs both the channel and the opening in the barrel wall, blocking the passage of the substrate to the lateral opening (Figure 2).

Exogenous LCFAs are first recognized at the surface of FadL by a low-affinity binding site formed by the two extracellular loops. Then, the LCFA diffuses to the high-affinity binding site within the channel. This LCFA translocation causes the displacement of the N-terminus plug, leading to the opening of the channel and the lateral release of the substrate in the OM (Figure 2) [9,10,11].

**Figure 3:**
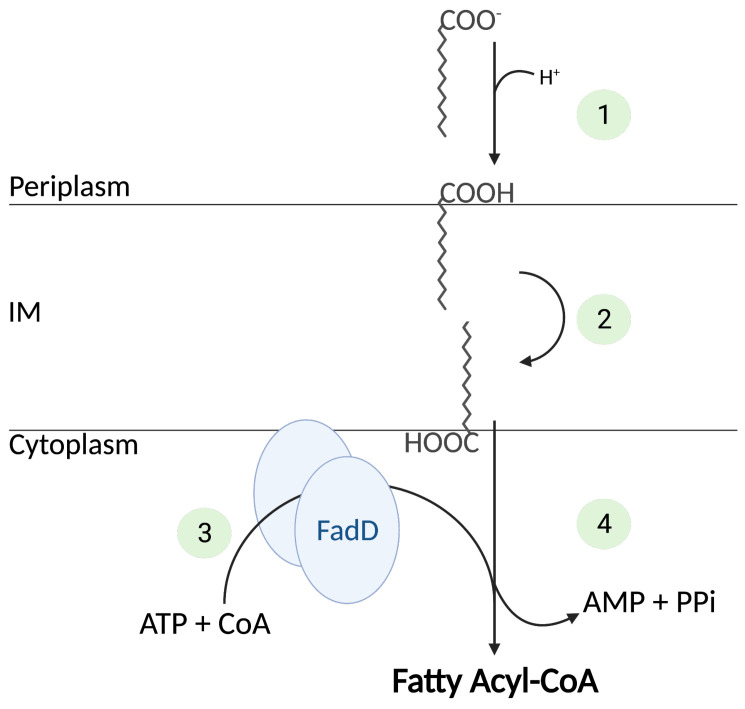
Vectorial transport and activation of FAs by FadD. Free FAs are protonated in the periplasm and then partitioned into the inner membrane (1). After flipping to the cytoplasmic face of the inner membrane (2), the free FAs recruit FadD proteins to the membrane (3), which extract them from the membrane concomitantly with their activation to acyl-CoA (4). Numbers correspond to the four steps required for the translocation and activation of the FA. IM = inner membrane, ATP = adenosine triphosphates; CoA = coenzyme A; AMP = adenosine monophosphates; PPi = pyrophosphate.

The mechanism of transfer of the LCFAs from the OM to the IM across the periplasm is still unclear. At the IM, LCFA crossing of the phospholipid bilayer is thought to occur via a flip-flop mechanism, followed by the vectorial thioesterification of the FA by FadD (Figure 3) [12,13]. The essential fatty acyl-CoA synthetase FadD, upon activation by ATP (adenosine triphosphate), couples the import of the LCFA with its activation by adding a coenzyme A molecule (CoA). FadD is a cytoplasmic homodimer of 62 kDa, which binds transiently to the IM cytoplasmic leaflet. LCFA activation consists of an energetically costly two-step reaction:

Fatty acid + ATP → acyl-AMP + PPi

Acyl-AMP + CoASH → acyl-CoA + AMP

The first step proceeds through the pyro-phosphorolysis of the ATP that catalyzes the acylation of the carboxyl group of the FA to the phosphoryl group of the AMP, while liberating a pyrophosphate; in the second step, the fatty acyl moiety is transferred to the sulfhydryl group of CoA, while the AMP is released [14].

Deletion of either *fadL* or *fadD* results in the inability of the cells to grow on FAs of any chain length as sole carbon sources. However, cells harboring a mutation in both *fadL* and *fadR* (encoding the repressor responding to LCFA, see Section 3.1) can grow on MCFAs, but not on LCFAs. This implies that the OM transporter FadL is specific for LCFAs, while FadD is able to process a broader spectrum of FAs [15,16,17].

Coupling of the transport and thioesterification of the FAs ensures that most FAs (98%) imported in the cell are in the form of fatty acyl-CoA thioesters. In this form, the fatty acyl-CoA thioesters are broken down through the β-oxidation cycle, where all the subsequent intermediates of the FA degradation pathway are thioesters linked to CoA (see below). Only 2% of the imported FAs within the cell are taken up by an acyl-ACP synthetase diverting this FA pool for the direct biosynthesis of phospholipids [18].

Interestingly, all the intermediates of FA degradation are thioesters of CoA, while all the intermediates of FA synthesis are thioesters of the small acyl carrier protein (ACP). This permits the separation of the degradation and synthesis pathways, which consist of similar but opposite reactions on otherwise identical substrates.

### 2.2. The β-Oxidation Cycle

#### 2.2.1. Degradation of Saturated Fatty Acids

The β-oxidation cycle by which FAs are degraded is conserved in prokaryotes and in the mitochondria of eukaryotes. After FA activation by the acyl-CoA synthetase FadD, the FA β-oxidation cycle involves four enzymatic activities (Figure 1): acyl-CoA dehydrogenase (ACD), 2-enoyl-CoA hydratase (ECH), 3-hydroxyacyl-CoA dehydrogenase (HACD), and 3-ketoacyl-CoA thiolase (KACT). At each cycle, the acyl-CoA is shortened by two carbons after the thiolytic cleavage releasing an acetyl-CoA. The degradation proceeds until the formation of a molecule of acetoacetyl-CoA that undergoes a last cleavage catalyzed by the thiolase II AtoB, related to the degradation of SCFA. At each turn of the β-oxidation cycle, one molecule of FADH_2_ (flavin adenine dinucleotide) and one molecule of NADH are produced by the ACD and HACD activities, respectively (Figure 1).

In *E. coli*, the four reactions of the β-oxidation cycle are carried out by three proteins: FadE, FadB, and FadA. FadE is a 89 kDa acyl-CoA dehydrogenase responsible for the first oxidative step of the cycle [19]. This reaction involves the transfer of two electrons from the substrate to the FAD cofactor. FADH_2_ must then be re-oxidized to regenerate the catalytic activity of the dehydrogenase. Apart from the characterization of its enzymatic activity, very little is known on the molecular aspects of FadE protein or its localization within the cell. It has been speculated that FadE may be able to function as an electron transfer flavoprotein thanks to an extra C-terminal domain (see Section 2.2.5), but to date, experimental support is lacking [19].

**Figure 4:**
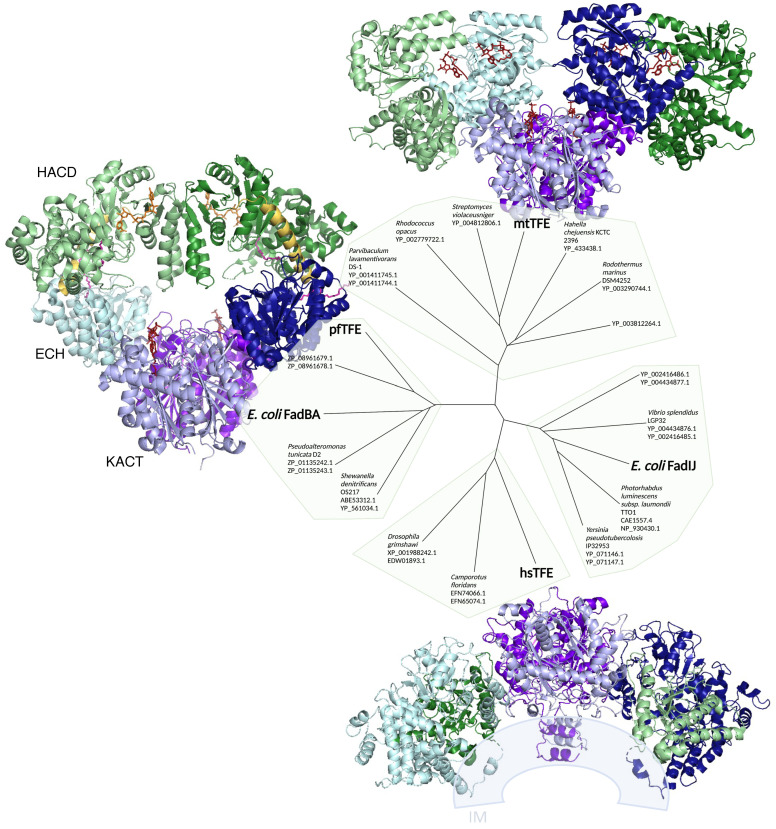
TFE complex families. TFE-a and TFE-b sequences have been proposed to group in four parallel subfamilies [24]. We are showing the pairs of TFE-a and TFE-b proteins in global families, indicated by the light shading on a phylogenetic tree widely simplified from [24]. The organisms for which the 3D structure of the TFE complexes have been studied are shown in bold letters. pfTFE = *Pseudomonas fragi* (PDB code: 1WDK) [23]; hsTFE = *Homo sapiens* (PDB code: 5ZQZ) [25]; mtTFE = *Mycobacterium tuberculosis* (PDB code: 4B3J) [24]. The quaternary assemblies of mtTFE, pfTFE, and mitochondrial hsTFE are shown anticlockwise from the top. The two TFEα ECH domains are shown in blue (light and dark); the two TFEα HACD domains are shown in green (light and dark). The TFEβ dimer (KACT domain) is at the center in violet (light and dark). Acyl-CoA is shown as red sticks. In the structure of pfTFE, the two α-helices linkers are shown in yellow, the NAD cofactors are shown as orange sticks, and the FA tail analogues in the TFEα are shown in pink. Predicted interaction of the hsTFE with the mitochondrial inner membrane (IM) is shown as suggested in [28].

**Figure 5:**
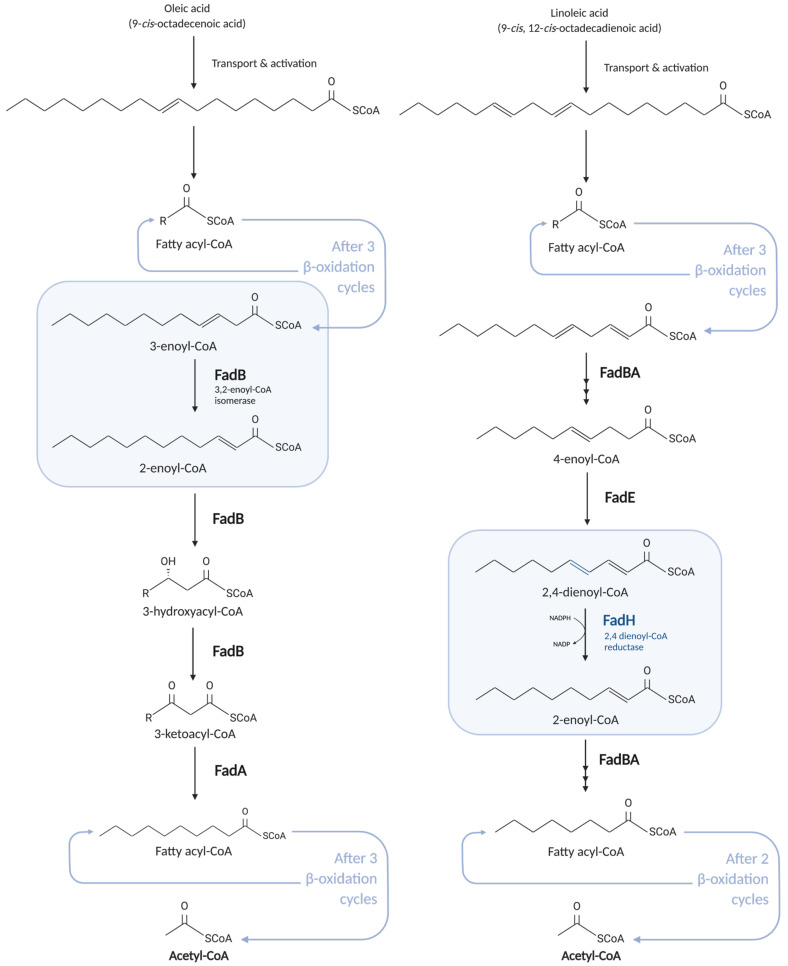
Degradation of unsaturated fatty acids (UFAs). UFA with a double bond extending from odd-numbered carbons (e.g., oleic acid, on the left) can be degraded thanks to the additional 3,2-enoyl-CoA isomerase activity of FadB (in the blue box on the left). UFA with double bonds extending from even-numbered carbons (e.g., linoleic acid, on the right) requires the auxiliary enzyme FadH carrying out a 2,4 dienoyl-CoA reductase activity (in the blue box on the right).

The enoyl-CoA generated by FadE is then the substrate for the three reactions carried out by the cytosolic tri-functional enzyme (TFE) complex FadBA (Figure 4) [20]. Four enzymatic activities are in fact associated with FadB: the enoyl-CoA hydratase (ECH) and 3-hydroxyacyl-CoA dehydrogenase (HACD) occur at each β-oxidation cycle (Figure 1). The two other additional activities performed by FadB, the *cis*-Δ^3^-*trans*-Δ^2^-enoyl-CoA isomerase and 3-hydroxyacyl-CoA epimerase, are required during the oxidation of unsaturated FA (UFA) (Figure 5) [21,22]. The saturated FA oxidation proceeds via three sequential reactions required to complete the cycle: the hydration and the oxidation steps performed by FadB, and the thiolytic cleavage carried out by FadA (Figure 1). The oxidative reaction carried out by FadB involves the transfer of electrons from the substrate to a NAD^+^ cofactor.

The TFE complex is well conserved in both mitochondria and bacteria. Three-dimensional structures have been obtained for bacterial and human TFE (Figure 4) [23,24,25]. In addition, the structure of FadB from *E. coli* was published recently [26]. The FadBA complex is a hetero-tetramer composed of two copies of the 78 kDa multifunctional enzyme FadB, and two copies of the 42 kDa 3-ketoacyl-CoA thiolase FadA. The mechanistic details of the channeling mode for the TFE FadBA complex have been uncovered [23]. In brief, the 3′-phosphate ADP moiety anchors the fatty acyl-CoA substrate to a common binding pocket in between the ECH and the HACD catalytic centers of FadB (Figure 4). This allows the fatty acyl-CoA group to pivot from the ECH to the HACD active site and then, toward the KACT active site of FadA. The incorporation of the acyl-CoA tail into the KACT hydrophobic cavity and the relocation of 3′-phosphate ADP bring the reactive C_2_–C_3_ bond of the FA to the correct position in the FadA active site for the thiolytic cleavage. This conformational transition weakens the ADP binding and detaches the substrate from the FadB pocket.

A channeling mechanism similar to that described for bacteria seems to be conserved in eukaryotes, as shown by the studies on the human mitochondrial TFE complex [25,27,28].

#### 2.2.2. Degradation of Unsaturated Fatty Acids

*E. coli* can also degrade unsaturated FA (UFA) with double bonds at even- or odd-numbered positions (Figure 5). For the degradation of UFAs with double bonds at odd-numbered carbons (e.g., oleic acid: *cis-*Δ^9^-octadecenoic acid), FadB carries out two additional reactions: *cis*-Δ^3^-*trans*-Δ^2^-enoyl-CoA isomerase and 3-hydroxyacyl-CoA epimerase activities, which allow the UFA to re-enter the β-oxidation cycle at the ECH step [29,30].

The catabolism of UFA with double bonds in even-numbered positions (for example, petroselinic acid: *cis*-Δ^6^-octadecenoic acid, or linoleic acid: *cis*,*cis*-Δ^9^,Δ^12^-octadecadienoic acid) requires an auxiliary enzyme to those of the β-oxidation cycle. Indeed, after a given number of cycles through the β-oxidation pathway, those UFAs contain 2-*trans,* 4-*cis* double bonds that cannot be further modified by FadB (Figure 5). In *E. coli*, the FadH auxiliary 2,4-dienoyl-CoA reductase overcomes this issue.

**Figure 6:**
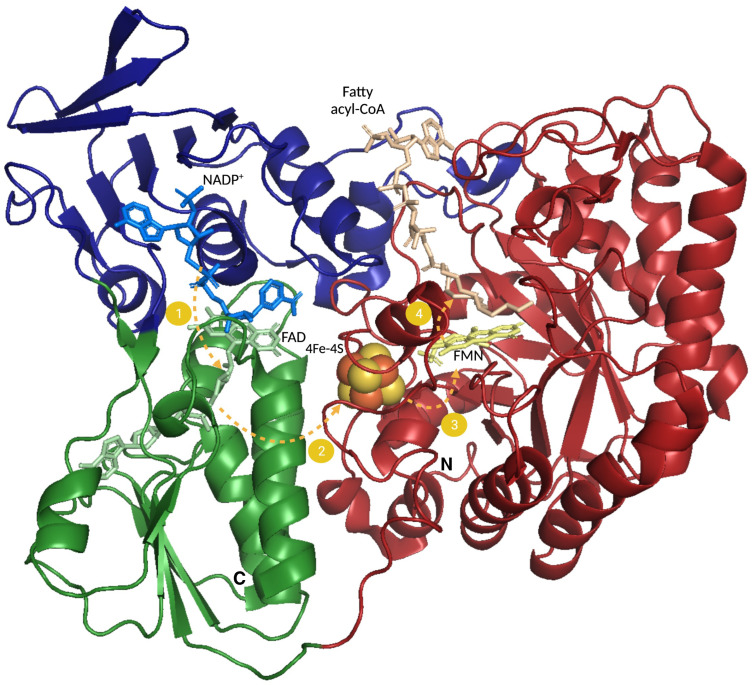
FadH structure. The 3D structure of *E. coli* FadH determined by X-ray crystallography is shown (PDB code: 1PS9) [35]. The N-terminal TIM barrel is colored in red, with the fatty acyl-CoA substrate in beige and FMN shown as yellow sticks. The [4Fe-4S] cluster is in the center of the figure (yellow and orange balls). The middle flavodoxin-like domain is in green, with FAD drawn as light green sticks. The C-terminal domain is in blue and includes NADPH, shown as light blue sticks. Yellow dashed arrows and numbers correspond to the electron flow during the reduction process from NADPH to the fatty acyl substrate. The N and C termini are indicated.

FadH is a 72 kDa monomeric protein that contains a [4Fe-4S] cluster, an NADPH, a FAD, and a flavin mononucleotide (FMN) domain (Figure 6) [31,32,33]. FadH uses the reducing power of an NADPH to remove the C_4_–C_5_ double bond of the 2,4-dienoyl-CoA substrate (Figure 5). It results in the formation of a 2-*trans*-enoyl-CoA product that can be incorporated again into the β-oxidation cycle. In this FadH-catalyzed hydride reaction, two reducing equivalents are directly transferred from NADPH to FAD, with a subsequent transfer of two electrons to FMN via the [4Fe-4S] cluster. The fully reduced FMN gives an ion to the C_5_ atom of the FA substrate, and the FadH active center residues protonate the C_4_ atom to complete the reaction [34,35].

Another type of UFA needs special attention. Indeed, although *E. coli* can fully degrade 90% of imported oleic acid, approximately 10% is converted into 3,5-*cis*-tetradecadienoyl-CoA that cannot be further degraded because the required enzyme, a dienoyl-CoA isomerase, is missing. Instead, the 3,5-*cis*-tetradecadienoyl-CoA is hydrolyzed to 3,5-*cis*-tetradecadienoic acid, which is then released in the medium. This hydrolysis is referred to as the thioesterase-dependent pathway of the β-oxidation cycle and it is essential to prevent the accumulation of minor metabolites which would otherwise inhibit the flux across the β-oxidation cycle [36].

*E. coli* possesses three thioesterases that could be involved in this thioesterase-dependent pathway: TesA (thioesterase I), TesB (thioesterase II), and FadM (predicted thioesterase III) [37]. Because TesA is a periplasmic protein, the cytosolic TesB and FadM proteins were the two candidates to carry out this additional reaction. Indeed, the growth on conjugated linoleic acid of an *E. coli tesB* and *fadM* double mutant was affected [38]. The double mutant was more impaired than the ∆*tesB* or ∆*fadM* single mutants. This suggested that both thioesterases may be involved and cooperate in the terminal hydrolytic reaction of the pathway [38]. Interestingly, FadM seems to be specifically involved in FA degradation since, in contrast with TesA and TesB, *fadM* is induced by the presence of LCFA and belongs to the *fad* regulon controlled by the repressor FadR (see §B.3) [37,39].

#### 2.2.3. S/MCFA Degradation

As the β-oxidation of the LCFA continues, the FA chain length progressively shortens to MCFAs and SCFAs, and the degradation of these FA species requires additional enzymes to be completed. S/MCFAs can also be directly acquired from the exogenous environment, especially in the gut.

MCFAs (C_10_–C_12_) are degraded by the core enzymes of the β-oxidation cycle (FadEBA). However, MCFAs with chain lengths shorter than C_14_ do not relieve FadR repression (see §B.3), so *E. coli* cannot use MCFAs as sole carbon and energy sources if LCFAs are not also present.

Interestingly, it has been shown that some natural and artificial strategies may come into play to allow the degradation of MCFA. Indeed, *E. coli* spontaneous mutants in *fadR* are readily isolated when growing on MCFA as the sole carbon source. Moreover, it was observed that the increase in the acyl-CoA pool in the cell, obtained by the artificial induction of the FadD synthetase, allows *E. coli* to counteract the FadR-mediated repression of the *fad* regulon and to grow on MCFA [40,41]. It revealed that *E. coli* FA degradation is not efficient. Indeed, during FA catabolism, *E. coli* accumulates short- and medium-chain thioester intermediates, as not all the FAs entering the β-oxidation are fully converted in the final acetyl-CoA product. This defect of *E. coli* seems to derive from the low efficiency of the FadEBA complex activity. Indeed, *Salmonella enterica* can completely convert the FAs to acetyl-CoA thanks to more efficient activities of the β-oxidation cycle [40]. This is surprising given the high sequence identity (>91%) of the FadEBA enzymes of *E. coli* and *Salmonella.*

**Figure 7:**
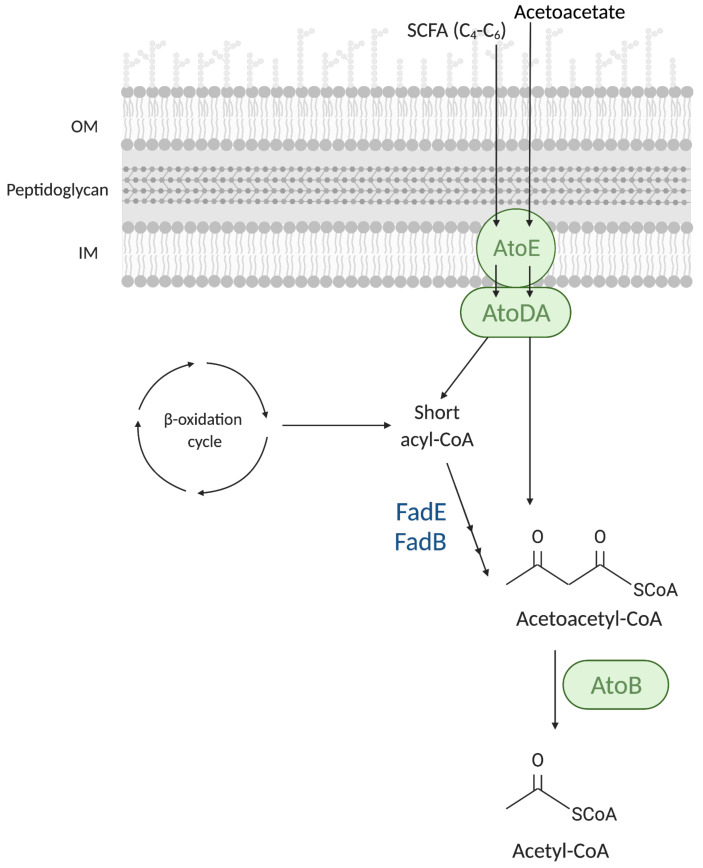
Degradation of short-chain fatty acids (SFCAs). AtoE is an inner membrane (IM) protein predicted to have ten transmembrane domains [43] involved in the import of SCFAs and acetoacetate across the IM. AtoD and AtoA are the α- and β-subunits of the acetyl-CoA:acetoacetyl-CoA transferase that activates the SCFAs and the acetoacetate by the CoA thioesterification. The catabolism of SCFAs requires an oxidation and a hydration step carried out by FadE and FadB, respectively. The AtoB thiolase II is in charge of the last thiolytic cleavage of the acetoacetyl-CoA to acetyl-CoA. The Ato enzymes specifically involved in the SCFA catabolism are shown in green capsules. FadE and FadB are indicated in blue.

The degradation of SCFA (C_4_–C_8_) in *E. coli* requires other enzymes in addition to those of the β-oxidation cycle (Figure 7). The AtoDAEB enzymes involved in the catabolism of SCFAs are induced by the presence of aceto-acetate (3-oxo-butanoic acid) [42]. Exogenous SCFAs presumably cross the OM via porin channels and diffuse across the cytoplasmic membrane in a non-ionized form [30]. AtoE is a membrane protein of the 2-hydroxycarboxylate transporter family [43] and is likely involved in SCFA import. The α- and β-subunits of the acetyl-CoA:acetoacetyl-CoA transferase, AtoD and AtoA, similarly to the step catalyzed by FadD, activate the acetoacetate to acetoacetyl-CoA [42,44]. Then, the 3-ketoacyl-CoA thiolase II, AtoB, catalyzes the thiolytic cleavage of the resulting short-chain fatty acyl-CoA to acetyl-CoA. In addition to the Ato enzymes, SCFA requires FadE and FadB enzymes to be fully degraded (Figure 7) [45]. For this reason, degradation of SCFAs in *E. coli* requires the presence of both acetoacetate and LCFAs, alleviating FadR repression of the *fad* regulon, to occur concurrently [30].

#### 2.2.4. The TCA Cycle and Glyoxylate Shunt

The tricarboxylic acid cycle (TCA) plays two essential metabolic roles in the cell. First, it allows the complete oxidation of acetyl-CoA during respiration [46]. It is highly regulated in the response of respiratory conditions. This is perfectly illustrated in facultative anaerobe bacteria such as *E. coli*, where the TCA cycle is an inducible pathway rather than a housekeeping process. Second, the TCA provides the required intermediates for the biosynthesis of several amino acids.

**Figure 8:**
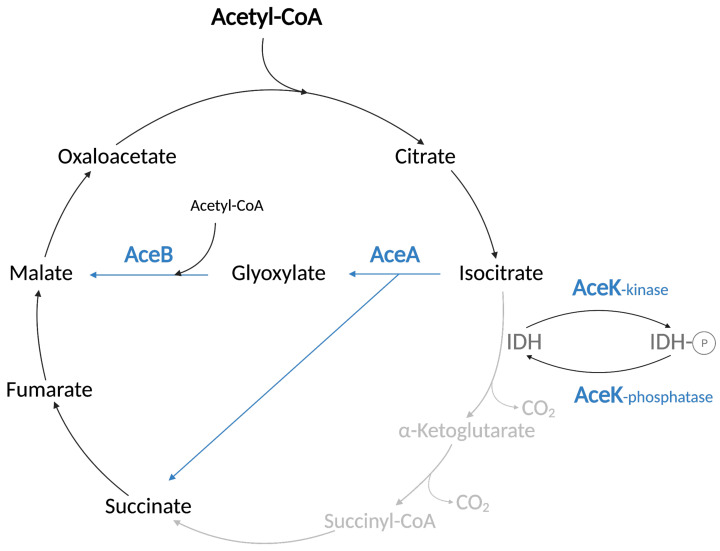
The glyoxylate shunt and related reactions. Acetyl-CoA enters in the tri-carboxylic acid (TCA) cycle. The isocitrate lyase AceA and the malate synthase AceB allow the conversion of isocitrate to malate and succinate by preventing loss of carbons in the form of CO_2_. The two reactions of the TCA cycle bypassed in the glyoxylate shunt are in gray. The regulation of the glyoxylate shunt by the AceK-dependent phosphorylation of the isocitrate dehydrogenase IDH is shown on the right. The IDH enzyme is shown in dark gray. All the intermediates of the cycle are in black. The enzymes involved in the glyoxylate bypass (AceABK) are in blue, as well as the arrows indicating the reactions carried out by AceA and AceB.

The glyoxylate shunt is essential for growth on substrates such as acetate or FAs that are degraded exclusively to acetyl moieties. Through a full TCA cycle, the acetyl-CoA would be quantitatively lost as CO_2_, and there would be no way to replenish the dicarboxylic acid pool necessary for the synthesis of amino acids and biomass production. The glyoxylate shunt resolves this problem. It contains five reactions of the TCA cycle, but it bypasses the two steps during which carbons are lost in the form of CO_2_ (Figure 8). The glyoxylate shunt requires three enzymes in addition to those shared with the TCA cycle. The isocitrate lyase AceA and the malate synthetase AceB convert the isocitrate substrate to malate and succinate (Figure 8). A bifunctional kinase/phosphatase AceK is required to decrease the activity of the isocitrate dehydrogenase of the TCA cycle that competes with the isocitrate lyase AceA for the isocitrate substrate (Figure 8). AceK inactivates the isocitrate dehydrogenase by phosphorylating the Serine 113 residue in the active site of the enzyme. This phosphorylation prevents the binding of isocitrate to the dehydrogenase [47]. The inactivation of the isocitrate dehydrogenase diverts the isocitrate substrate from the TCA cycle to the glyoxylate shunt, resulting in the formation of a branch point between the two metabolic pathways during growth on FAs. Importantly, the three enzymes AceA, AceB, and AceK are induced during growth on FAs (see Section 3.1).

#### 2.2.5. Fatty Acid β-Oxidation and the Respiratory Chain

Growth on FAs requires the presence of either oxygen as final electron acceptor (in aerobic condition) or alternative electron acceptors such as nitrate (in anaerobic condition) to allow reducing equivalents to be re-oxidized through respiratory chains. Indeed, FAs are a non-fermentable carbon source, and therefore, they require a functional electron transfer mechanism to channel the reducing power produced by the β-oxidation cycle (one molecule of FADH_2_ and one NADH are produced at each round) to the components of the electron transfer chain (ETC) located in the IM. In addition, the growth on LCFA has been suggested to increase the oxidative stress in *E. coli* more than the growth on other non-fermentable carbon sources (i.e., acetate or glycerol), due to the accumulation of reduced cofactors produced during the β-oxidation cycle [48]. Therefore, an optimal coupling between the β-oxidation cycle and the respiratory ETC is needed. In mitochondria and in bacteria, electron transfer flavoproteins (ETF) are involved in the transfer of electrons from flavoenzymes and dehydrogenases to the respiratory chains. ETFs are two-subunit enzymes (composed of ETFα and ETFβ chains). In mitochondria, they are required as a hub to take up the electrons from flavoenzymes and dehydrogenases, and to feed them into the respiratory chain. Amino acid catabolism, FA oxidation, and choline metabolism are among the metabolic routes in mitochondria in which ETFs intervene as electron transfer components [49]. In bacteria, ETFs are involved in similar types of catabolism. For example, in *Mycobacterium tuberculosis*, the EtfDAB enzymes are required for growth on FAs [4]. Most bacterial ETFs are homologous to the FixB and FixA proteins found in nitrogen-fixing and diazotrophic bacteria.

In *E. coli*, no ETF system required specifically during growth on FAs has been identified so far. Three sets of ETF homologs are found in *E. coli*. The FixAB proteins are required for anaerobic carnitine reduction [50]. The YgcRQ and YdiQR proteins are of unknown function. YdiQR were proposed as candidates for growth on FAs in anaerobic conditions due to the proximity of the genes with the acyl-CoA synthetase *fadK* gene [51], but experimental evidence is lacking.

An ETF activity was initially proposed to be associated with the FadE acyl-CoA dehydrogenase. Interestingly, the FadE enzyme is 814 residues long, two times the size of the mammalian counterpart. The sequence alignment between FadE and the mammalian enzyme shows the presence of additional domains of 150 and 400 residues at the N-terminal and the C-terminal ends of FadE, respectively [19]. The essentiality of the FadE C-terminus was shown in *Salmonella* *enterica* serovar Typhimurium [52]. When the C-terminal domain of the *S. enterica* FadE was deleted, the enzyme lost its biological activity in vivo. Therefore, the *E. coli* FadE C-terminus was speculated to work itself as a flavoprotein domain. After the FAD reduction to FADH_2_, FadE C-terminus was supposed to be involved in the transfer of electrons from the FadE central dehydrogenase domain to the respiratory chain in order to re-generate the reducing power [19,51]. However, there is no experimental evidence to support this role of FadE yet.

### 2.3. Diversity of the Fad Enzymes

#### 2.3.1. TFE Diversity in Bacteria

The bacterial β-oxidation machinery was first discovered and characterized in *E. coli* [12]. However, in the past two decades, the study of FA degradation has been extended to several microorganisms and highlighted a wide degree of diversity both in the Fad enzymes and the substrates metabolized. The multiplicity of the *fad* genes uncovered in Gram-positive and Gram-negative bacteria underlines the biological relevance of the FA degradation in the central carbon metabolism of microorganisms. Moreover, the ability to degrade a broad range of different FAs provides bacteria with the competitive advantage to adapt to different niches and environments. For example, *Pseudomonas putida* displays two β-oxidation machineries (β_I_ and β_II_) that can degrade alkanoic and phenylalkanoic acids, but to different extents and efficiencies. Whereas the β_I_ (FadAB1) is constitutively expressed, the β_II_ (FadBA5) is induced when the genes of the first system are mutated, such as to allow the bacteria survival [53]. *Pseudomonas aeruginosa* genome contains five FadBA pair homologs. The FadBA5 pair was shown to be involved in LCFA utilization and to be induced by LCFA [54,55]. In *M. tuberculosis,* a genomic survey revealed the presence of several FadA-like thiolases whose physiological functions are not currently known. The identified thiolases were classified into four families depending on the presence and length of an extra helix (LA5), and on their genetic linkage, or not, with genes encoding an associated TFE-α chain, acyl-CoA dehydrogenases, or enoyl-CoA hydratases/isomerases. One of these additional TFE-β, FadA2, was found to be more closely related to the human TFE-β subunit (35% identity) than to the mycobacterial FadA1 (27% identity), and to not interact with the TFE-α [24]. In a last example of multiple β-oxidation systems, *Streptomyces coelicolor* has three FadBA complexes whose expression vary in relation with the stage of growth and the presence of FA in media [56].

Therefore, the existence of multiple homologs of the *fad* genes in different bacteria matches the great chemical diversity of FAs occurring in nature. This diversity strongly supports the key participation of the respective enzymes in the bacterial fitness, possibly linking FA metabolism to the exploitation of new niches, adaptation to nutritional challenges, or still unidentified bacterial processes.

#### 2.3.2. A Second TFE in *E. coli*: The FadIJ Proteins

*E. coli* contains homologs of FadB and FadA, called FadJ and FadI, respectively. Snell and coworkers observed that an *E. coli* mutant in the *fadB* gene was still able to produce PHA from the β-oxidation of LCFA in an engineered strain [57]. They identified the homologous FadJ protein and further showed that it was responsible for the residual activity. Moreover, the *fadJ* gene is in operon with *fadI*, coding for a FadA-like thiolase. FadIJ and FadBA sequences are about 35% identical. This suggested that the homologous FadIJ enzymes might carry out the FA β-oxidation at the place of the canonical pair FadBA [57]. In addition, *fadIJ* genes were shown to be induced by LCFA in a FadR-dependent manner [51,58]. The year after, the group of Dr. Cronan observed that *E. coli* was still able to grow slowly on LCFAs in a *fadA* mutant, whereas the double mutant *∆fadA* ∆*fadIJ* was not [51]. In addition, growth of the ∆*fadA* mutant was more robust in the absence of oxygen. Thus, the authors suggested that *fadIJ* might be specifically involved in FA β-oxidation in the absence of oxygen. However, the double mutant ∆*fadIJ* grew perfectly well on LCFA in anaerobiosis. Therefore, the core machinery (FadBA) seems to be the main machinery required for FA degradation, independent of oxygen availability, whereas the second pair, FadIJ, is likely required in case of defects in the main system [51].

Despite their homology, in vitro analysis suggested that FadIJ and FadBA complexes present many different features [26,59]. FadIJ was modeled as a hetero-octameric enzyme, possibly membrane-bound. Moreover, FadIJ was proposed to prefer M/LCFA, while FadBA preferentially metabolizes SCFA. In fact, FadIJ TFE shares several common properties with the human TFE (HsTFE), and phylogenetic analysis confirmed that the α and β subunits of the FadIJ are more closely related to HsTFE than to the FadBA complex (Figure 4) [24,59]. Based on these in vitro data, and on the mitochondrial model, Sah-Teli and colleagues speculated that the membrane-bound FadIJ system carries out the β-oxidation cycle of very long- and long-chain FAs, and then the soluble system, FadBA, intervenes to catalyze the reactions on shorter acyl-CoA [59]. This model contrasts the alleged role of FadIJ in the degradation of FA in the absence of oxygen and the specificities of FadBA and FadIJ hypothesized by the group of Dr. Cronan [51]. This discrepancy further suggests that the understanding of the interplay between the FadBA and FadIJ complexes in the FA degradation has yet to be untangled.

#### 2.3.3. Acyl-CoA Synthetase Diversity in Bacteria

The diversity of the acyl-CoA synthetases, coupling the FA translocation and activation, is even wider than the one observed for the TFE complexes. For instance, six FadD enzymes have been described in *P. aeruginosa.* Two were shown to differentially contribute to FA degradation, with one being specific for LCFA and the second for SCFA [60]. Another one, FadD4, was found to be involved in the degradation of FAs and plant-derived acyclic terpenes, citronellic, and geranic acids [61]. Even more synthases were identified in actinobacteria such as *M. tuberculosis*, which is predicted to encode 36 FadD-like enzymes, likely involved in the catabolism of structurally diverse FA within human macrophages [62,63].

#### 2.3.4. The Acyl-CoA Synthetase FadK

*E. coli* FadK was proposed to be an acyl-CoA synthase homolog to FadD as they share 29% identity and 57% similarity, with a conservation of the typical AMP binding site and the acyl-CoA synthase motif [64].

An *E. coli* ∆*fadD* mutant is completely blocked in the aerobic β-oxidation and therefore cannot grow on LCFA. However, a *fadD* mutant was shown to grow on S/MCFA and poorly on LCFA in the absence of oxygen, suggesting the presence of an alternative acyl-CoA synthase used in the absence of oxygen [51]. FadK was a good candidate for this role and, indeed, a double mutant ∆*fadD* ∆*fadK* failed to use FA of all chain lengths as carbon source either aerobically or anaerobically. Nonetheless, a single *fadK* mutant grows robustly on all FAs, independently from oxygen levels, thanks to FadD activity [51]. The authors further showed in vitro that FadK had low activity on L/MCFA and was maximally active on SCFA (C_6_ and C_8_) [64]. Given the essentiality of FadD for the β-oxidation, regardless of oxygen levels present, these observations rather suggest different and maybe complementary substrate specificity between the two acyl-CoA synthetases FadD and FadK, rather than a specific role of FadK in the absence of oxygen (see also Section 3.3).

## 3. Genetic Regulation of Fatty Acid Degradation in *E. coli*

**Figure 9:**
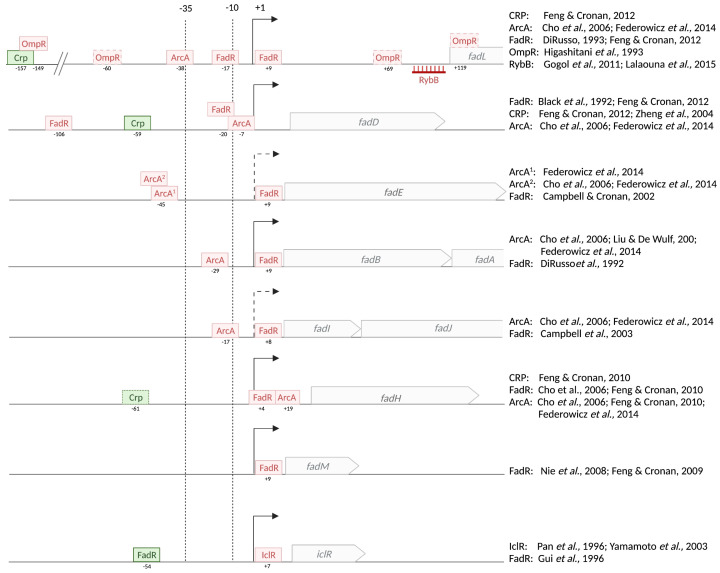
Position of the transcriptional regulator binding sites in *fad* promoters. The promoter transcription start sites are indicated by arrows (+1). The −10 and −35 regions are aligned and marked by the dotted vertical lines. The dashed arrows for *fadIJ* and *fadE* indicate the prediction from whole-genome determination of transcription start sites [65]. The other transcription start sites were determined as described in the references indicated below. The position of each binding site is marked by boxes with the name of the corresponding transcriptional regulator. The distance of the middle of each binding box to the transcription start site is given below the box. The repressors are shown in red, and the activators of gene expression in green. The position of hybridization of the sRNA RybB on FadL mRNA is also indicated. The references for specific studies of the expression of the indicated gene are the following: *fadL*: [66,67,68,69,70,71,72]; *fadD*: [67,68,69,73,74,75]; *fadE*: [19,68,69]; *fadBA*: [68,69,76,77]; *fadIJ*: [51,68,69,78]; *fadH*: [68,69,79]; *fadM*: [37,39]; *iclR*: [80,81,82].

The genes coding for the fatty acid degradation enzymes are scattered around the chromosome of *E. coli* and hence transcribed independently (Figure 9). Only the *fadBA* and *fadIJ* gene pairs are transcribed as operons, reflecting the tight association of the polypeptides that form the trifunctional enzyme (cf. Section 2.2; Figure 4). Interestingly, *fadIJ* genes are located at the same locus as *fadL*, but in reverse orientation. This genetic link with the outer membrane FA importer strengthens the role and importance of *fadIJ* in fatty acid degradation, even though no obvious phenotype of a *fadIJ* mutant has been evidenced yet.

Fatty acids are not the preferred carbon and energy sources for the cell. Moreover, they may be only transiently available to enterobacteria depending on the diet intake. Therefore, the expression of *fad* genes must be precisely controlled. *fad* genes are under tight control of the FadR repressor responding to the presence of FAs. They are also controlled by the global cAMP/CRP system, avoiding the use of FAs when a better carbon source is available. Finally, as most genes involved in the catabolism of alternative carbon sources, the genes are repressed in the absence of oxygen by the ArcAB two-component system.

### 3.1. Regulation by Substrate Availability: The Transcriptional Repressor FadR

**Figure 10:**
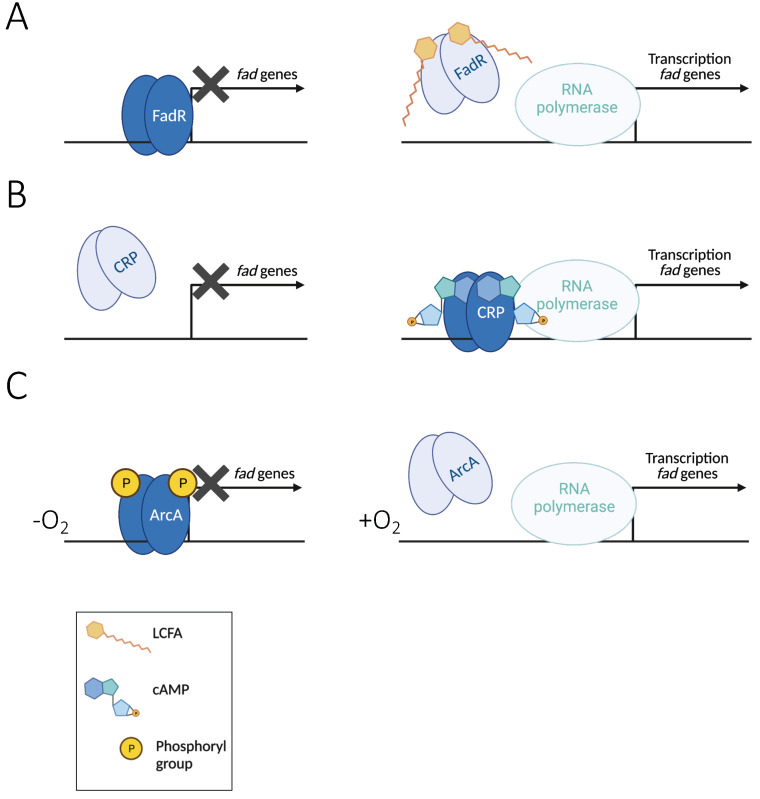
Control of *fad* gene expression by FadR, CRP, and ArcA. (**A**) FadR. A dimer of FadR binds the *fad* promoters. It prevents the RNAP binding and inhibits the transcription, as shown by the black cross (**left**). Binding of long-chain acyl-CoA results in conformational modification of FadR, which leaves the DNA, and as a consequence, the RNAP is recruited and transcription starts (**right**). (**B**) CRP: catabolite repression in the presence of glucose prevents *fad* gene expression (**left**). In the absence of glucose, the cAMP levels increase and the cAMP/CRP dimer binds the DNA and recruits the RNAP, so the transcription starts (**right**). (**C**) ArcAB two-component system: the absence of oxygen is sensed by ArcB, which auto-phosphorylates and transfers the phosphoryl group to ArcA. ArcA-P binds to DNA, preventing the RNAP recruitment (**left**). In the presence of oxygen, ArcA cannot bind DNA and the *fad* genes are transcribed (**right**). A legend with the cofactors of the different regulators is shown in the panel on the bottom.

The expression of the genes involved in fatty acid degradation is increased when exogenous long-chain fatty acids are available. This control is performed by the transcriptional repressor FadR. *fadR* mutants were identified long ago as gain of function mutants able to grow on short-chain FA (shorter than 12 carbons), whereas wild-type *E. coli* cannot [12]. This was later explained by the fact that *fadR* codes for a repressor of FA degradation genes, and that this repression is relieved by the binding of acyl-CoA containing 14 carbons or more [76] (Figure 10A).

**Figure 11:**
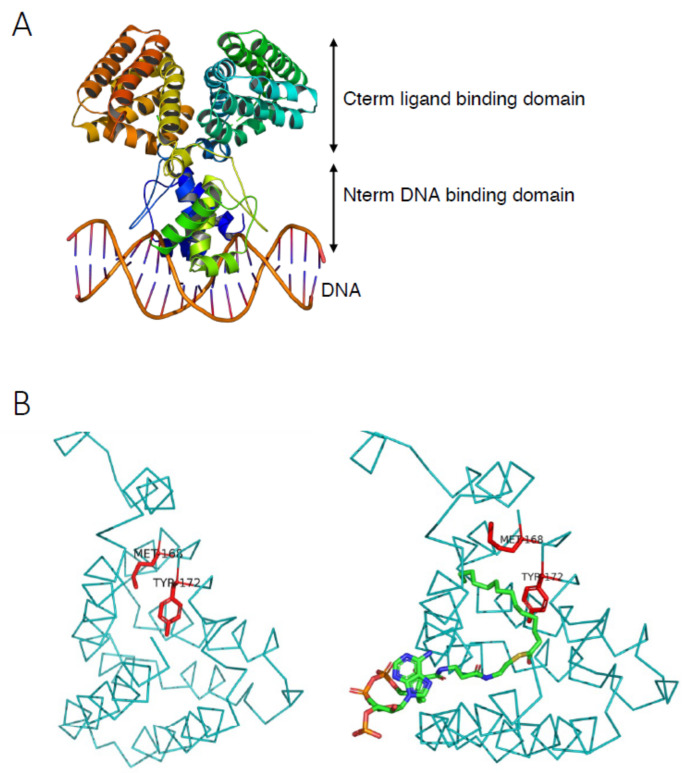
Structure of the FadR regulator. (**A**) Apo-FadR dimer bound to the DNA operator. PBD ID: 1H9T. (**B**) Close-up of the ligand binding domain. Apo-FadR on the left (PBD ID: 1E2X) and myristoyl-CoA bound on the right (PBD ID: 1H9G). The methionine 168 and tyrosine 172 residues are indicated in red, to highlight their movement upon ligand binding [85,86].

*E. coli* FadR is a 239-residue protein of the GntR regulator family and functions as a dimer (Figure 11). The FadR N-terminal domain binds to DNA thanks to a typical winged-helix motif, while the C-terminal domain is the acyl-CoA ligand binding domain [83].

#### 3.1.1. FadR DNA Binding Features

Mutagenesis experiments permitted the dissection of the DNA binding mechanism [5,84]. Since then, 3D structures of the apo-FadR dimer bound to DNA have permitted to understand in detail the recognition between FadR and DNA [83,85,86] (Figure 11A). Depending on the *fad* gene, one or two FadR binding sites overlap with the transcription start site, which prevents the access of the promoter to RNAP (Figure 10A).

**Figure 12:**
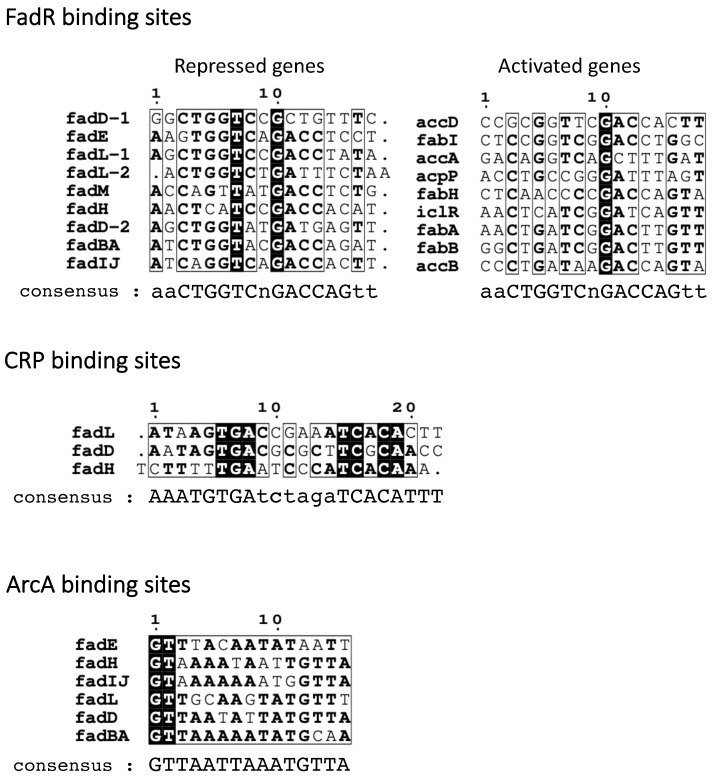
Binding sites of FadR, CRP, and ArcA in *fad* promoters. The binding site sequences were retrieved from the references given in Figure 9. The consensus sequences were retrieved from the Ecocyc website [107]. The alignments were formatted using the ESPrit 3.0 server [108]. Strictly conserved and more than 60% conserved nucleotides are highlighted in black and bold, respectively.

The small number of FadR target genes described initially led to the definition of a weak DNA binding consensus sequence for FadR in *E. coli* [66,87]. The identification of new target genes has permitted the description of the binding site as a 17 pb palindrome aaCTGGTCnGACC(T/A)Gtt (Figure 12). Interestingly, comparative genomics approaches have permitted refining this consensus in diverse orders of gamma-proteobacteria [88]. Lastly, the study of the FadR–DNA binding specificities by the selection of random oligonucleotides led to a very similar consensus sequence [80].

#### 3.1.2. FadR Ligand Binding Properties

Long-chain fatty acyl-CoA (≥C_14_) are the specific ligands of *E. coli* FadR [76]. Acyl-CoA interacts directly with the FadR C-terminal domain. The acyl-chain is buried deep inside a hydrophobic pocket (Figure 11B). This binding provokes conformational changes of the Met168 and Tyr172 residues inside the pocket that must rotate to accommodate the ligand. This movement induces a large rigid body movement, modifying the distance between the DNA recognition helices of the two monomers, which alters DNA binding [86]. Mutagenesis experiments have permitted the dissection of the FadR ligand binding site in detail [5,89]. Using this information, it was then possible to obtain constitutive mutations that render the FadR variant insensitive to the addition of FA [89,90].

If the size limit for *E. coli* FadR de-repression is 14 carbons or more, it has been shown that increasing the intracellular pool of acyl-CoA by overproducing FadD or by expressing heterologous FadR protein can decrease the size limit in *E. coli* [40,41]. FadR from various bacteria display variable affinities and specificities in the size limit of detection of acyl-CoA molecules [91]. For instance, FadR of *Vibrio cholerae* possesses a 40-residue insertion that creates a second FA binding site for each monomer [91,92]. This may explain the observed higher sensitivity of this protein to the presence of FA. Strikingly, ligand binding to *Vibrio* FadR provokes a huge movement in the structure, much more extensive than the one observed in FadR from *E. coli*. This variation in the mode of ligand binding is consistent with the fact that FadR proteins from diverse γ-proteobacteria present a wide range of FA binding affinities, with the *Vibrio* FadR having the highest [91].

Interestingly, the specific response of FadR to the presence of FA makes it a useful biotechnological tool for engineering strains to produce chemicals or fuels derived from fatty acids or for the design of biosensors [93]. FadR biosensors have also been described to screen genes increasing the fatty acyl-CoA pool in *Saccharomyces cerevisiae* [94] or for the design of a fluorescent sensor enabling the quantification of fatty acyl-CoA in living human cells and subcellular compartments [95]. Since FadR from various bacteria display different affinities and specificities for different acyl-CoAs [91], and since it has been shown that it is possible to fine-tune FadR binding capacity by site-directed mutagenesis [90], there is leverage for engineering FadR proteins to respond to specific FAs.

#### 3.1.3. The FadR Regulon

FadR directly represses the expression of all the actors of FA degradation (*fadL*, *fadD*, *fadE*, *fadBA*, *fadIJ*, *fadH*, *fadM*) (Figure 9). The binding site is generally overlapping the −10/+1 region of the promoters of the genes.

As the first line in the detection of exogenous fatty acids, the *fadL* and *fadD* genes are somewhat not as strongly repressed by FadR as the other *fad* genes, despite the presence of two binding sites for FadR [67,73]. Curiously, *fadD* promoter exhibits two FadR binding sites. The position of the second site at −100 from the transcription start site is surprising if it is to act as a repressor. No in vivo experiments have been performed to assess the importance of this site for *fadD* control. In contrast, the positions of FadR binding sites within *fadL* [67], *fadE* [19], *fadBA* [76], and *fadIJ* [51] promoters are consistent with a repressing activity as they overlap the transcription start sites.

*fadM* and *fadH,* although required only for the degradation of certain types of FA, are induced in the presence of any LCFA through FadR de-repression [39,79]. It means that even if their preferred substrate is not present, they will be induced in the same way as the other core Fad enzymes by LCFA.

FadR is a dual regulator as it can also act as an activator of transcription. In this case, it binds upstream of the −35 region from the transcription start site in order to help RNAP binding. Among the genes activated by FadR is *iclR* [80]. IclR is itself a repressor of the *aceBAK* operon coding for the enzymes of the glyoxylate shunt. Therefore, in the presence of LCFA, *iclR* expression is reduced and *aceBAK* genes are activated. Indeed, as explained before, growth on fatty acids requires the glyoxylate shunt to permit the entry of the acetyl-CoA products of the β-oxidation into the TCA cycle (cf. Section 2.2).

Importantly, FadR plays a dual role in the regulation of lipid metabolism. In addition to *iclR*, it is also an activator of nearly all fatty acid biosynthesis genes. Indeed, it was recognized early that FadR activates the expression of the *fabA* and *fabB* genes involved in the biosynthesis of unsaturated fatty acids [96,97,98]. Yet, more recently, it was shown that FadR modulates the expression of all *fab* genes [99,100]. Therefore, the presence of LCFA will not only activate the expression of Fad degradation genes, but it will also slow down the expression of FA synthesis genes. Hence, FadR is a transcriptional factor that positively regulates the anabolism and negatively regulates the catabolism of the same family of molecules [66]. It was also the first example of repression mediated by a positive activator [97]. This dual role of FadR in FA degradation and synthesis seems specific to *E. coli*, as in other bacteria, two distinct regulators are commonly present to solve the two functions. Note that, unlike many other DNA binding proteins, FadR does not autoregulate its synthesis [97,101].

A transcriptome study of the FadR regulon in the presence or absence of FAs has permitted researchers to discover the activation of *fabB* by FadR [98], then to identify the gene coding for FadE [19], and finally to discover the genes coding for FadIJ proteins [51]. The reverse approach of a transcriptome study of a strain overproducing FadR has been documented [93]. However, in this report, transcriptomic and proteomics experiments were performed in an engineered strain (a ∆*fadE* mutant expressing artificially a cytoplasmic acyl-CoA thioesterase), so the effects might not be reflecting physiological conditions.

### 3.2. Regulation by Carbon Source Availability: The CRP/cAMP Activator

The *fad* regulon is strongly repressed in the presence of glucose, and the presence of LCFAs cannot relieve this repression [12,13,102]. However, when glucose gets low, cAMP starts accumulating, and two cAMP molecules bind the cAMP receptor protein (CRP) dimer, which can then interact with the consensus sequence on the DNA and recruit the RNAP [74,103] (Figure 10B).

The cAMP/CRP complex positively regulates the expression of several genes of the *fad* regulon [67]. cAMP/CRP binding sites have been described in the promoters of *fadL*, *fadD*, and *fadH*, and these genes are upregulated in limited glucose conditions, suggesting direct binding of the cAMP/CRP protein to these promoters [67,74,79] (Figure 9 and Figure 12). More recently, a CRP binding site was identified in the promoter of the *fadIJ* operon, explaining its upregulation during carbon limitation [78] (Figure 9 and Figure 12). *fadBA* gene expression was also described to be downregulated in a *crp* mutant; however, no binding site was identified [67,74]. The control of *fadE* expression by cAMP/CRP has not been investigated specifically, but the induction of acyl-CoA dehydrogenase activity coded by *fadE* was shown to be coordinated with the other FA degradation enzymes [102], and *fadE* was identified in a whole-genome study of CRP regulon [78], suggesting that it is certainly also affected by cAMP/CRP, potentially indirectly, as was proposed for *fadBA*.

Interestingly, the *fad* regulon appears to be upregulated in diverse conditions of stress, especially upon starvation or entry in the stationary phase. A first observation was the upregulation of the *fadB*, *fadL*, and *fadD* genes during growth arrest [87]. This phenomenon was further studied for *fadD* and proposed to be part of a mechanism of “emergency de-repression” [104]. This de-repression was shown to involve the cAMP/CRP regulation, and this might be a general mechanism explaining the de-repression of *fad* genes in the absence of exogenous FAs. Consistently, the *fad* regulon was repeatedly found upregulated during growth arrest in more recent studies, often in correlation with CRP control [78,105]. In addition to cAMP/CRP, regulation by FadR might have another level of control of *fad* genes in the stationary phase. Indeed, *fadR* expression was shown to be repressed by the growth-related ppGpp global regulator, and the quantity of FadR protein was lower in the stationary phase [99]. The decrease in FadR might contribute to the upregulation of *fad* genes in the stationary phase. Finally, in an *E. coli* pathogenic strain, the *fad* genes were shown to be upregulated by the global stress response regulator RpoS that is involved in reprogramming gene expression in the stationary phase [106]. While heterogenous in context, all these studies point to a role of *fad* genes during growth arrest that might be important for membrane homeostasis control.

### 3.3. Repression in Anaerobiosis by the ArcAB Two-Component System

The *fad* genes are also regulated by the ArcAB two-component system. ArcAB is a global regulatory system that responds to the redox state of the cell, also dependent on the oxygen level [109]. ArcB is a membrane-associated tripartite sensor kinase comprising a transmitter domain, a receiver domain, and a phosphotransferase domain, whereas ArcA is the transcriptional regulator sensu stricto. A reduction in oxygen concentration induces a change in the quinone pool in the inner membrane, which in turn triggers the autophosphorylation of ArcB, which phosphorylates ArcA. Phosphorylated ArcA binds the target promoter region and represses the expression of many genes [109] (Figure 10). In the absence of oxygen, the phosphorylated ArcA binds to the promoter regions of *fadL*, *fadD*, *fadE, fadBA*, and *fadH* [68] (Figure 9 and Figure 12). The joint repressions by FadR and by ArcA have been shown to result in additive effects on *fadL, fadD*, and *fadH* genes, and synergistic effects on *fadBA* expression [67,68,79].

Interestingly, *fadIJ* genes are also repressed by ArcA and negatively regulated by FadR [51,68]. FadIJ proteins have been proposed to function as an alternative pathway for FA degradation in the absence of oxygen [51] (see Section 2.3). However, subsequent studies on the regulation of these genes have clearly shown that they are controlled exactly like the *fadBA* homologous genes (expressed in presence of oxygen and LCFAs). Importantly, they are repressed in anaerobiosis by the ArcAB two-component system, which brings some doubt regarding a specific role in anaerobiosis (see Section 2.3.2). Yet, as with the other *fad* genes, *fadIJ* can be derepressed in the absence of oxygen if LCFAs are present.

FadK has also been proposed to act in FA degradation in anaerobiosis (see Section 2.3.4). However, increased expression of *fadK* in anaerobic conditions has not been observed in transcriptome studies [69,75], which is in contradiction with the observed increase reported previously [64]. Furthermore, there is no evidence of the regulation of *fadK* by FadR or CRP, another argument suggesting that further studies are required to ascertain the role of FadK in FA degradation either in the presence or absence of oxygen.

### 3.4. Regulation of fadL by Stress Response

As the outer membrane receptor and importer of FAs from the environment, FadL is in first line and can therefore be additionally tuned to control the induction of the *fad* genes, before the entrance of FAs inside the cell and the FadR response. It is therefore not so surprising that the *fadL* gene is controlled by additional stress response pathways. First, *fadL* is repressed by the osmoregulator EnvZ/OmpR two-component system [70]. Four potential OmpR binding sites have been evidenced by foot-print experiments in *fadL* promoter (Figure 9), resulting in the repression of *fadL* expression at high osmolarity [70]. In hyperosmotic conditions, several other outer membrane transporters such as the OmpC and OmpF porins are regulated by the EnvZ/OmpR two-component system. In these conditions of cell shrinkage and dehydration, the control of FA import might be important to maintain cell integrity.

*fadL* is also repressed by the alternative sigma factor σ^E^, in response to envelope stress. This is due to direct repression by the small RNA RybB, itself induced by σ^E^ [71]. The interaction between *fadL* mRNA and RybB has been further confirmed by global MS2-sRNA affinity purification [72] and shown to require the Hfq RNA chaperone [110].

## 4. Open Questions and Research Needs

We have described here the role of the β-oxidation machinery of *E. coli* for growth on FAs as the sole carbon source. However, it is possible that the Fad machinery might also be involved in other roles than exogenous FA consumption. Indeed, it has been proposed that in the stationary phase, free FAs are released due to the recycling of the envelope and its phospholipids, and that these FAs are processed by the Fad enzymes [6,7]. This certainly must be put in relation with the upregulation of *fad* genes in the stationary phase, and the involvement of the different global regulators ppGpp, RpoS, and CRP should be assessed within this context. Moreover, outer membrane stress can be detected by a phospholipase, and the FAs produced act as signaling molecules requiring the function of the acyl-CoA synthase FadD [2]. The multiplicity of Fad enzymes observed in *E. coli* and other bacteria might play a role in these stress response conditions.

The Fad isoforms may also show different substrate specificities yet to be discovered. Research on this aspect is important for the development of new metabolic engineering strategies. Indeed, FA metabolism is being used widely to produce biofuels and derived molecules of interest, and the manipulation of the FA β-oxidation enzymes are key in this engineering [111,112,113].

The biochemistry of the β-oxidation pathway in *E. coli* is well known. In contrast, there is no information about the molecular organization of the proteins in the cell: Does the stable TFE FadBA complex interact with the other components such as FadD and FadE, or the accessory enzymes FadH and FadM that are all produced at the same time upon FadR de-repression? Another crucial question is to know how the reducing power generated by the β-oxidation pathway is transferred to the respiratory chain. If FadE is involved as it has been proposed, we need structure/function and biophysical studies on the mechanism of FadE, to understand what the exact function of the FadE C-terminal domain is. Finally, is there a specific localization of the enzymes in the cell? FadD is expected to be associated with the inner membrane for the import of the FAs [114], and it has been proposed that the FadIJ complex might also be bound to the membrane [59]. A localization of the machinery at the membrane might help in transferring the reductive power to the respiratory chain.

In addition, these studies on the molecular and cellular aspects of the Fad enzymes might help to clarify the proposed role of the FadIJ and FadK enzymes in a specific anaerobic degradation system.

The *fad* genes of *E. coli* are controlled by a common complex network of regulators (FadR, CRP, and ArcAB described in this review). This regulation is now well described and understood, yet some gaps remain: the expression of the *fadE* gene has not been studied much, and how FadR and ArcA can be both repressors by binding to rather distant binding sites is unclear. A thorough clarification of the transcription start site(s) and binding experiments is needed. Moreover, all the *fad* genes are repressed by catabolite repression, yet the direct CRP control has been shown only for *fadD*, *fadL*, *fadIJ*, and *fadH* genes (Figure 9). It will be important to determine if the control of the other *fad* genes by cAMP/CRP is direct or not, and if indirect, through which mechanism.

In addition to the FadR, CRP, and ArcAB regulators controlling *fad* gene expression in response to metabolic clues, there is certainly an additional fine-tuning of the expression of *fad* genes by stress response regulators. This is exemplified by the regulation of *fadL* by the OmpR regulator depending on osmolarity [70], and by the small RNA RybB activated during envelope stress [71], or by the upregulation of *fad* genes during the stationary phase. Given the complexity of the regulatory network of the *fad* genes, it would not be surprising that an additional layer of regulation by sRNA intervenes. Interestingly, two recent reports have shown the involvement of sRNA regulation on *fad* genes. In *V. cholerae*, the sRNA FarS reinforces the repression of the two *fadE* genes by FadR [115]. In *E. coli*, a new sRNA co293 inside the e14 prophage seems to repress *fadR* expression [116].

To conclude, numerous examples of the contribution of FA metabolism to bacterial infections, either for signaling or for FA degradation, suggest that this metabolism plays a crucial role in responding to the host environment. Investigating the wide chemical diversity of FAs along with the multiple FA degradation pathways evolved by bacteria will be especially important to assess the contribution of FA metabolism to bacterial multiplication and survival in natural settings.
